# Intersectionality matters for Hispanic health: A replication study using the All of Us Research Program

**DOI:** 10.1186/s12939-024-02280-7

**Published:** 2024-09-30

**Authors:** Mariana Rodrigues, Emma Risner, Brennan Rhodes-Bratton, Stephanie H. Cook, Adolfo Cuevas

**Affiliations:** 1 Department of Social and Behavioral Sciences, New York University School of Global Public Health, 708 Broadway, 4th floor, New York, NY USA; 2 Department of Biostatistics, New York University School of Global Public Health, New York, NY USA; 3 Center for Anti-racism, Social Justice, and Public Health, New York University School of Global Public Health, New York, NY USA

**Keywords:** Intersectionality, Hispanic health, Racial and ethnic differences, Health disparities

## Abstract

**Background:**

Despite research dedicated to understanding the health profiles and health-related outcomes of Hispanic individuals, the prevailing body of literature frequently homogenizes the Hispanic population, failing to address the role of race in Hispanic health discourse. Thus, the current study applies an intersectional lens to identify health differences and similarities among Hispanic subgroups.

**Methods:**

Sociodemographic characteristics and health domain variables (i.e., health status, health services, and health behaviors) from participants (*N* = 11,192) were included in the analyses. Bivariate Chi-squared tests examined the relationship between sociodemographic and health domain variables Black Hispanic individuals, white Hispanic individuals, and non-Hispanic Black individuals.

**Results:**

Findings suggest that Non-Hispanic Black American individuals reported the highest rates of hypertension (49.09%) and diabetes (19.62%) compared to Black-Hispanic individuals (22.45% and 12.98%) and white Hispanic individuals (22.22% and 8.02%). Black Hispanic individuals reported the greatest proportion of asthma diagnoses (35.10%) and those who saw a doctor in the previous year (95.52%) compared to white Hispanic individuals (26.84%, and 91.10%, respectively) and non-Hispanic Black individuals ( 21.74%, and 94.69%, respectively).

**Conclusion:**

Specifically, we found that several health behaviors and health-related outcomes significantly varied across different racial/ethnic groups, demonstrating the advantage of an intersectional approach to identify health disparities among racially diverse ethnic groups.

**Public Health significance:**

We encourage the development of health care services with an awareness of the complexities resulting from racial differences within the Hispanic diaspora.

## Background

The Hispanic/Latino population (hereafter Hispanic) is the largest minority group in the United States (US). Projections indicate that their representation could reach 29% of the US population by 2050 [1]. Despite robust research dedicated to understanding the health profiles of Hispanic individuals, the prevailing body of literature frequently homogenizes the Hispanic population, failing to address the role of race in Hispanic health discourse.

Over the years, many authors and policymakers have argued about the conceptualizations of race and ethnicity. It is critical to highlight, however, that racial and ethnic definitions depend on location, class, and nationality [2, 3]. For Hispanic individuals, for instance, the term ethnicity refers to “the array of values, cultural norms, and behaviors of the different subgroups share”, while race “represents categorization of individuals on the basis of skin color, both by self-identification and by the eye of the beholder” [4].

According to intersectionality theory, multiple marginalized identities intersect and interact with existing power structures creating unique experiences of discrimination and oppression [5, 6]. This lens is essential to understand how race and ethnicity intersect and interact with power dynamics, shaping individuals’ health, behaviors, and access to services. In the context of health in the United States, for instance, Borrell [4] developed a conceptual framework to better explain the effect of race on Hispanic individuals and its implications. In summary, the framework posits that first, Hispanic individuals categorize themselves into U.S. Census racial categories based on societal perceptions. This racial categorization directs Hispanic individuals toward different opportunities and disadvantages. These factors, interacting with social structure such as segregation, environmental exposure, racism, and discrimination, influence health outcomes dynamically over a lifetime, with negative experiences potentially impacting future generations. For instance, Black Hispanic individuals potentially face the similar health consequences faced by non-Hispanic Black individuals due to multiple risks and social disadvantages such as stress and social isolation stemming from systemic and structural racism. As such, racial categorization among Hispanic subgroups could be a significant predictor of health status and well-being [4].

Emerging research has revealed distinct health outcomes and behaviors between Black and White Hispanics [7]. Borrell & Dallo [8], for instance, investigated the association between race and self-rated health among Hispanic and non-Hispanic individuals using data from the National Health Interview Survey (NHIS; 2000–2003) [9] and found that non-Hispanic Black individuals had the highest prevalence of poor self-rated health compared to their counterparts.

More recently, LaVeist-Ramos [10] and colleagues harnessed eight years of data from the NHIS to create a substantial cohort of Black Hispanics individuals. They found the health behaviors of Black Hispanic individuals mirror those of white Hispanic individuals (i.e., both Black and white Hispanic individuals were more likely to be current drinkers in comparison to non-Hispanic Black individuals), while health service outcomes (i.e., having a usual source of care and having seen a doctor within the past year) align more closely with non-Hispanic Black individuals [10]. Notably, the health status of Black Hispanic individuals emerges as an intricate interplay of both race and ethnicity, as they exhibit health behaviors and health service outcomes profiles similar to both white Hispanic and non-Hispanic Black adults (i.e., diabetes, heart condition, and stroke prevalence estimates) [10]. The health effects of racism and the deep-rooted influence of culture may influence different health profiles for Black and white Hispanic individuals. The well-being of Black Hispanic individuals can oscillate, leaning more towards either non-Black Hispanic or white Hispanic individuals, depending on these social determinants.

The work by LaVeist-Ramos and team [10] began to untangle these complex threads that set race apart from ethnicity. Nonetheless, it is critical to note that study findings derived from the NHIS, which relies on self-reported data susceptible to recall bias and is predominantly conducted in English, potentially limiting participation among non-English speakers. To enhance the credibility of LaVeist-Ramos findings [10] replication is crucial. The utilization of the *All of Us* [11] initiative offers a rich opportunity to examine the health profile among non-Hispanic Black Americans, Black Hispanic Americans, and white Hispanic Americans. Contextually, the *All of Us* program, a National Institute of Health’s initiative, is one of the largest and diverse biomedical datasets of its kind [11]. Significantly, the *All of Us* initiative has made strides in recruiting historically underrepresented adult samples. With over 40,000 Hispanic enrollees, the *All of Us* Research program is the largest cohort of Hispanic individuals in the United states [12] with surveys available both in English and Spanish, thus affording an unprecedented opportunity to replicate the LaVeist-Ramos findings with a representative cross-section of the Hispanic population.

This replication study is a pivotal step forward, enabling us to view these health differences and similarities among Hispanic subgroups through an intersectional lens. Understanding of how race and ethnicity intersect within the Hispanic population, allows us to develop tailored interventions that take into account the intricate interplay of these factors, ensuring that healthcare strategies and policies address the unique needs of different Hispanic subgroups. Recognizing and examining intersectionality within the context of race and ethnicity among Hispanics is a vital first steps toward achieving health equity and improving overall well-being. In addition, by exploring potential differences and similarities between Hispanic individuals with non-Hispanic Black individuals, we seek to highlight the unique and shared experiences among these groups as a better understanding of the different and interconnected roles race and ethnicity might play as predictors of health and well-being.

## Methods

### Data and participants

This replication study employs a cross-sectional design using data from the *All of Us* Research Program, funded by the National Institutes of Health. The *All of Us* Research Program is a US biomedical research initiative that uses an array of health information from electronic health records (EHRs), wearables, surveys, physical measurements, and genome sequencing. Further details about the program are outlined extensively elsewhere [4]. *All of Us* contains data from 409,420 participants, however; only 11,192 participants were included on the conditions of belonging to the Black Hispanic, white Hispanic, or non-Hispanic Black ethnic/racial groups, identifying as male or female, and having at least one non-missing answer to the presence of the health conditions in order to replicate the parent study [10]. This research was approved through the *All of Us* Research Project registration process.

### Measures

#### Demographics

**Age**. The age of the participants was ascertained by subtracting their date of birth from the survey date time. The remainder of the demographic variables were ascertained via self-reporting in the baseline survey.

**Race/Ethnicity**. Race/ethnicity was ascertained via self-reporting when participants were asked to identify which racial and ethnic categories best describe them. Participants selected all racial and ethnic categories that applied to their identity from the following list: American Indian or Alaska Native, Asian, Black/African American, Hispanic/Latino/Spanish, Middle Eastern or North African, Native Hawaiian or other Pacific Islander, White, None of these, or Prefer not to answer. If a participant indicated Hispanic/Latino/Spanish and white, they were recategorized as white Hispanics. If Hispanic/Latino/Spanish and Black/African American were selected, the participants were recategorized as Black Hispanic individuals. Participants who indicated Black/African American race with no indication of Hispanic/Latino/Spanish were categorized as non-Hispanic Black Americans. All other racial/ethnic groups and missing data were excluded from the analysis.

**Gender**. The gender selection of the participants was limited to those who answered as male or female to reproduce the original study. Thus, participants who identify as non-binary, transgender, or who selected additional options were dropped from the study. Those who indicated they preferred to avoid answering or skipping the question were considered missing.

**Income**. Income was self-reported with possible values less than $10k, $10k- $25k, $25k- 35k, $35k- 50k, $50k- 75k, $75k- 100k, $100k- 150k, $150k- 200k, more than $200k, and prefer not to answer. To reproduce the parent study, the income levels were collapsed into $0–34,999, $35,000–74,999, and $75,000 and higher. Those who indicated they preferred not to answer or skipped the question were considered missing.

**Education**. Education was reported as the highest- level of education earned: Less than 9th grade, 9th grade through 12th grade, high school diploma or GED, some college, college graduate, advanced degree, and prefer not to answer. To duplicate the previous study, college graduates and advanced degrees were combined into one category: college graduates. Those who indicated they preferred not to answer were considered to be missing.

**Marital Status**. Marital status was reported as married, never married, divorced, living with a partner, widowed, separated, and preferred not to answer. Divorced and separated were combined into one category: living with a partner and married. Those who indicated they chose not to answer were considered to be missing.

**Nativity**. Participants were asked in what country they were born and indicated if they were born in the US, foreign-born, or preferred not to answer. Those who preferred not to respond or skipped the question were considered missing.

#### Health domains

**Health Status**. The personal and family health history survey captured all health status questions. Participants self-reported who had at least one non-missing answer to the following health status questions were included in the final analysis. Participants indicated as having ‘no matching concept’ or who elected to skip the question were considered missing in each health status variable. Hypertension and stroke were assessed through the circulatory conditions’ domain; the binary variable indicating the history of hypertension and/or stroke was derived. Diabetes was assessed with a binary variable indicating a doctor diagnosis of type 1 or type 2 diabetes or not. Asthma was assessed with a binary variable indicating a doctor’s diagnosis of the condition or not. Any heart condition was obtained through the circulatory conditions’ domain, and those with atrial fibrillation, congestive heart failure, coronary artery conditions, history of heart attack, heart valve disease, pulmonary embolisms, or an unlisted heart condition were considered as having a heart condition and combined into a singular binary variable ‘any heart condition.’

**Health Services**. The insurance variable is a binary variable determined by if the participant identified as having insurance coverage with the question “Are you covered by health insurance or some kind of health care plan?” with possible answers of ‘yes,’ ‘no,’ and ‘don’t know’ in the baseline survey. Those who answered as ‘don’t know’ were considered missing. The usual source of care is a binary variable determined by the health care access and utilization survey question “Is there a place that you usually go to when you are sick or need advice about your health?” with possible answers of ‘yes,’ ‘more than one,’ ‘no,’ ‘don’t know.’ Participants who indicated they did have a usual source of care or more than one source were categorized as ‘yes,’ while those who answered ‘don’t know’ were considered missing. “Seen a doctor in the past year” was determined by the question “About how long has it been since you last saw or talked to a doctor or other health care provider about your own health?” in the health care access and utilization survey. Participants who indicated they had seen a doctor within the past 12 months were considered ‘yes,’ while those who reported a longer period than 12 months were considered ‘no.’ Lastly, participants who indicated they did not know or skipped the question were considered missing.

**Health Behaviors**. Three health behaviors were measured for the study: weight, tobacco smoking status, and alcohol consumption. The four weight categories (underweight, healthy, overweight, and obese) were created from the numeric body mass index (BMI) value. Underweight participants were indicated by a BMI less than 18.5. Healthy weight participants had a BMI between 18.5 and 24.9. Overweight participants reported a BMI value between 25 and 29.9. Obese participants had BMI values of 30 and above. Two questions in the lifestyle survey determined tobacco smoking status: (1) “Have you smoked at least 100 cigarettes in your entire life? (There are 20 cigarettes in a pack.)?” and (2) “Do you now smoke cigarettes every day, some days, or not at all?”. Current smokers were considered those who reported currently smoking, while never smokers reported not currently smoking and had not smoked more than 100 cigarettes in their lifetime. Those who reported they didn’t know, preferred not to answer, or skipped the question were considered missing. Two questions from the lifestyle survey determined alcohol consumption: (1) “In your entire life, have you had at least 1 drink of any kind of alcohol, not counting small tastes or sips? (By a “drink,” we mean a can or bottle of beer, a glass of wine or a wine cooler, a shot of liquor, or a mixed drink with liquor in it.)”; (2) “How often did you have a drink containing alcohol in the past year?”. Those who indicated they have ever had a drink in their life and drank it at the frequency of 2–3 drinks per week, 2–4 drinks per month, and 4 or more drinks per week were considered current drinkers. Those who indicated a frequency of monthly or less or never were not regarded as current drinkers. Those who answered, don’t know, preferred not to answer or skipped the questions were considered missing.

### Statistical analysis

All analyses were conducted using R Statistical Software (v4.2.2) [13] in the *All of Us* workbench. The final participant sample data was stratified by the three racial/ethnic groups (white Hispanic individuals, Black Hispanic individuals, and non-Hispanic Black individuals), and univariate statistics were calculated. Frequencies and proportions were reported for categorical variables, and for the continuous variable, age, the mean and standard error (SE) were reported. Bivariate Chi-squared tests were employed to examine the relationship between demographic and health domain variables among the racial/ethnic groups. Additionally, a t-test was used to compare differences in the mean age among the racial/ethnic groups. Specifically, comparisons were examined for Black Hispanic individuals to white Hispanic individuals and Black Hispanic individuals to non-Hispanic Black individuals.

## Results

Table 1 displays the frequency and proportions for categorical demographic variables and the mean and standard error (SE) for age stratified for each race category. Additionally, the significant findings of the chi-squared tests (*p* < .05) and the t-tests (*p* < .05) are indicated.

**Table 1:** Select demographics by race/ethnicity among adults ≥ 18 years of age among the all of us dataset (*N* = 11,192)

Variable	Black Hispanic *N* = 208 (1.86%) *N* (%)^1^	White Hispanic *N* = 1833 (16.38%) *N* (%)^1^	Non-Hispanic Black *N* = 9151 (81.76%) *N* (%)^1^
**Age** (mean [SE])	40.72 [0.93]	41.40 [0.36]	50.53 [0.15] †
**Gender**			
Male Female	50 (24.27%) 156 (75.72%)	559 (30.63%) 1266 (69.37%)	2251 (24.78%) 6834 (75.22%)
**Education**			
Less than 9th Grade 9th-12th High School/GED Some College College Graduate	1 (0.48%) 1 (0.48%) 34 (16.75%) 86 (43.35%) 79 (38.92%)	7 (0.39%) 36 (2.04%) 164 (9.05%) * 502 (27.69%) * 1103 (60.84%) *	93 (1.03%) 516 (5.73%) † 1807 (20.07%) 2985 (33.15%) † 3604 (40.02%)
**Income**			
$0–34,999 $35,000–74,999 $75,000+	94 (52.22%) 46 (25.56%) 40 (22.22%)	443 (26.17%) * 449 (26.52%) 801 (47.31%) *	4344 (54.68%) 1981 (24.94%) 1619 (20.38%)
**Marital Status**			
Never Married Married/Living as Married Divorced/separated Widowed	85 (42.71%) 80 (40.20%) 31 (15.58%) 3 (1.51%)	572 (31.45%) * 1003 (55.14%) * 213 (11.71%) 31 (1.70%)	3372 (37.76%) 2929 (32.80%) † 2133 (23.88%) † 497 (5.56%) †
**Nativity**			
US Born Foreign Born	169 (81.25%) 39 (18.75%)	1535 (84.06%) 291 (15.94%)	8455 (93.21%) † 616 (6.79%) †

Note: *p*-values are by the χ2 statistic for gender, education, income, marital status, nativity and language of interview and by the t statistic for age. GED; general equivalency diploma

^1^ mean (SE) reported for continuous variables

*p-values (< 0.05) for comparisons of black Hispanics with white Hispanics

†*p*-values (< 0.05) for comparisons of black Hispanics with non-Hispanic blacks

Table 2 provides the frequencies and proportions for each health domain variable and indicates the significant findings of the chi-squared tests comparisons between the ethnic-racial groups. Non-Hispanic Black Americans reported the greatest proportion of hypertension (49.09%), followed by Black Hispanic Americans (22.45%) and white Hispanic Americans (22.22%). The chi-squared tests indicated a significant difference in the proportion of those with hypertension in the non-Hispanic Black American group versus Black Hispanic Americans. Similarly, the racial/ethnic group with the greatest proportion of those with diabetes were non-Hispanic Black Americans (19.62%), Black Hispanic Americans (12.98%), and finally white Hispanic Americans (8.02%). These chi-squared tests indicated a significant difference in the proportions of those with diabetes for white Hispanic Americans versus Black Hispanic Americans and non-Hispanic Black Americans versus Black Hispanic Americans. Non-Hispanic-Black Americans reported the greatest proportion of any heart condition (16.98%), followed by white Hispanic Americans (15.42%) and Black Hispanic Americans (14.29%). Non-Hispanic Black Americans reported the greatest percentage of having experienced a stroke (2.81%), followed by Black Hispanic Americans (1.92%), and white Hispanic Americans (0.93%). Black Hispanic Americans reported the greatest proportion of asthma (35.10%), followed by white Hispanic Americans (26.84%) and non-Hispanic Black Americans (21.74%). The chi-squared tests indicated a significant difference in asthma proportions between Black Hispanic Americans and white Hispanic Americans and for the non-Hispanic Black Americans versus the Black Hispanic Americans.

**Table 2:** Select health domain demographics by race/ethnicity among adults ≥ 18 years of age among the all of us dataset (*N* = 11,116)

Variable	Black Hispanic *N* = 208 (1.86%) *N* (%)^1^	White Hispanic *N* = 1833 (16.38%) *N* (%)^1^	Non- Hispanic Black *N* = 9151 (81.76%) *N* (%)^1^
**Health Status**			
Hypertension Diabetes Any Heart Condition Stroke Asthma	44 (22.45%) 27 (12.98%) 28 (14.29%) 4 (1.92%) 73 (35.10%)	392 (22.22%) 147 (8.02%) * 272 (15.42%) 17 (0.93%) 492 (26.84%) *	4128 (49.09%) † 1795 (19.62%) † 1478 (16.98%) 257 (2.81%) 1989 (21.74) †
**Health Services**			
Insurance Coverage Usual Source of Care Seen a doctor in past year	193 (94.61%) 178 (90.82%) 192 (95.52%)	1756 (96.64%) 1619 (92.41%) 1627 (91.10%) *	8260 (92.04%) 8161 (93.16%) 8381 (94.69%)
**Health behaviors**			
**BMI**			
Obese Overweight	95 (52.20%) 64 (35.16%)	549 (40.10%) * 371 (27.10%) *	4673 (59.23%) 1958 (24.82%) †
**Smoking Status**			
Current smoker Never smoker	59 (29.65%) 140 (70.35%)	514 (25.43%) * 1294 (71.57%) *	3135 (34.78%) 5879 (65.22%)
**Drinking Status**			
Current drinker Never drinker	188 (91.26%) 18 (8.73%)	1749 (95.73%) * 78 (4.27%) *	7858 (86.88%) 1187 (13.12%)
**Activity Level**			
Vigorous activity	10 (55.56%)	195 (56.20%)	431 (55.68%)

Note: *p*-values are by the χ2 statistic

*p-values (< 0.05) for comparisons of black Hispanics with white Hispanics

†*p*-values (< 0.05) for comparisons of black Hispanics with non-Hispanic blacks

White Hispanic Americans reported the greatest proportion of insurance coverage (96.64%), followed by Black Hispanic Americans (94.61%) and non-Hispanic Black Americans (92.04%). Non-Hispanic-Black Americans reported the greatest proportion of participants who had a usual source of care (93.16%), followed by white Hispanic Americans (92.41%) and Black Hispanic Americans (90.82%). Black Hispanic Americans reported the greatest proportion of those who saw a doctor in the past year (95.52%), followed by non-Hispanic Black Americans (94.69%) and white Hispanic Americans (91.10%). The chi-squared tests indicated a significant difference in the proportion of white Hispanic Americans versus Black Hispanic Americans who saw a doctor in the past year.

Non-Hispanic Black Americans reported the greatest proportion of those with an obese BMI (59.23%), followed by Black Hispanic Americans (52.20%) and white Hispanic Americans (40.10%). The chi-squared tests indicated a significant difference in proportions of obese participants between white Hispanic Americans versus Black Hispanic Americans. Black Hispanic Americans reported the highest proportion of those with an overweight BMI (35.16%), followed by white Hispanic Americans (27.10%) and non-Hispanic Black Americans (24.82%). The chi-squared tests indicated a significant difference in the proportions of overweight participants between white Hispanic Americans versus Black Hispanic Americans and between non-Hispanic Black Americans and Black Hispanic Americans. Non-Hispanic Black Americans reported the highest proportions of current smokers (34.78%%), followed by Black Hispanic Americans (29.65%) and white Hispanic Americans (25.43%). White Hispanic Americans reported the highest proportion of current drinkers (95.73%), followed by Black Hispanic Americans (91.26%) and non-Hispanic Black Americans (86.88%). The chi-squared test indicated a significant difference in the proportion of current drinkers in the white Hispanic Americans versus the Black Hispanic Americans. Lastly, white Hispanic Americans reported the greatest proportion of those who participated in vigorous activity (56.20%), followed by non-Hispanic Black Americans (55.68%) and Black Hispanic Americans (55.56%).

## Discussion

Our results highlight significant disparities in health behaviors and health service outcomes among racial and ethnic groups. We used an intersectional approach to shed light on the differences in health behaviors and health-related outcomes and services among Black Hispanic individuals, white Hispanic individuals, and non-Hispanic Black individuals. We found that race and ethnicity have independent associations with health behaviors and health-related outcomes.

Non-Hispanic Black individuals had higher rates of self-reported hypertension, diabetes, obesity, and having experienced any heart condition and a stroke when compared to Black Hispanic and white Hispanic individuals as well as reported the highest proportion of being current smokers. Black Hispanic individuals had higher self-reported asthma diagnoses, and highest proportion of those with overweight compared to non-Hispanic Black Americans and white Hispanic individuals. Finally, white Hispanic individuals reported the highest proportion of being current drinkers and participating in vigorous activity compared to non-Hispanic Black Americans and Black Hispanic individuals.

In addition, our results show that white Hispanics reported the greatest proportion of insurance coverage, followed by Black Hispanic individuals and non-Hispanic Black Americans. On the other hand, Black Hispanic individuals had the highest reporting rates of those who saw a doctor in the past year, followed by non-Hispanic Black Americans and white Hispanic individuals. Finally, non-Hispanic Black Americans reported the greatest proportion of participants who had a usual source of health care.

In contrast to the results reported by LaVeist-Ramos and colleagues [10], where ethnicity (Hispanic) appeared to play a predominant role in health behaviors, our findings indicate that race (identifying as Black) plays a more dominant influence on health behaviors and health-related outcomes. In particular, we found that race is associated with health outcomes among Hispanic individuals, in that Black Hispanic individuals report elevated rates of smoking, asthma diagnoses, and overweight compared to their white Hispanic counterparts. This corroborates earlier findings from smaller-scale studies, which have reported that Black Hispanic individuals exhibit higher body mass index, elevated rates of smoking, and increased asthma prevalence when compared to their white Hispanic counterparts [10, 14]. These findings suggest that Black Hispanic individuals have significantly heightened risk of disease compared to their white counterparts. Failing to recognize this disparity, and instead viewing Hispanic individuals as a monolithic group, could potentially mask the urgent health challenges faced by Black Hispanic individuals.

The current study notably contributes and expands on the existing literature by showing that the intersection of race and ethnicity could result in additional barriers to health-related services. For instance, our results showed that Black Hispanic individuals had the highest reporting rates of those who saw a doctor in the previous year while not having the highest insurance coverage. To our knowledge, no research has explored the health profiles of Black Hispanic individuals in the U.S. healthcare system. Thus, there is a pressing need to consider the intersection of race and ethnicity in health service delivery.

The limitations of our analysis are also factors future research critically needs to explore. First, the health behaviors and health-related outcomes are based on self-reported data, which introduces potential bias. Future research should use objective measures (e.g., biological markers) to further explore health differences and similarities among Hispanic subgroups. Additionally, for the purposes of this replication study, we only considered the sex-assigned at birth of participants. As such, those who identified as non-binary, transgender, or selected additional options were not considered for the analysis. By doing so, we did not explore differences in health behaviors, health-related outcomes, or services of racial/ethnic sexual and gender minorities. Future research should investigate such differences across racial and ethnic groups to develop interventions that are accessible and inclusive of those at the intersection of multiple minoritized identities who experience increased health inequities and barriers to health care. Finally, due to the replication nature of this study, we conducted only bivariate analyses. Future research should conduct further analyses to examine which factors may explain differences in health by race and ethnicity. By doing so, researchers will incorporate an intersectionality framework and have a better understanding of the ways in which minoritized identities, in addition to race and ethnicity, intersect and interact with systems of oppression, thus leading to a disproportionate impact on the health and well-being of Hispanic individuals.

## Conclusion

Racialization in the US has a profound impact on health, as evidenced by the disparities in health status between non-Hispanic Black Americans and Black Hispanic individuals when compared to white Hispanic individuals. The divergence in the health profiles of Black Hispanic from those of non-Hispanic Black Americans may indicate the presence of compensatory factors that partially mitigate the adverse effects of racism on health for Black Hispanic individuals. However, it is important to note that this mitigation is not absolute, as Black Hispanic individuals still exhibit compromised health behaviors and outcomes in specific areas. To gain a better understanding of these disparities and the underlying factors, future research should examine the root causes of these health inequities by utilizing an intersectionality approach. Additionally, it is important to investigate the psychosocial resources that are common and distinctive among these groups, shedding light on the complex interplay of race and ethnicity in existing health differences.

## Data Availability

Data for this study were drawn from the All of Us Research Program, funded by the National Institutes of Health (https://allofus.nih.gov/).
